# High-Density Horizontal Stacking of Chondrocytes via the Synergy of Biocompatible Magnetic Gelatin Nanocarriers and Internal Magnetic Navigation for Enhancing Cartilage Repair

**DOI:** 10.3390/polym14040809

**Published:** 2022-02-19

**Authors:** Shan-Wei Yang, Yong-Ji Chen, Ching-Jung Chen, Jen-Tsai Liu, Chin-Yi Yang, Jen-Hao Tsai, Huai-En Lu, San-Yuan Chen, Shwu-Jen Chang

**Affiliations:** 1 Department of Orthopedics, Kaohsiung Veterans General Hospital, Kaohsiung City 813414, Taiwan; yangshanwei@yahoo.com.tw; 2 Department of Biomedical Engineering, I-Shou University, Kaohsiung City 813414, Taiwan; ygchen2021@gmail.com (Y.-J.C.); eddy3264@gmail.com (C.-Y.Y.); harelove0423@gmail.com (J.-H.T.); 3 School of Opto-Electronic Technology, University of Chinese Academy of Sciences, Beijing 100049, China; cjchen@ucas.ac.cn; 4 College of Materials Science and Opto-Electronic Technology, University of Chinese Academy of Sciences, Beijing 100049, China; jtliu@ucas.ac.cn; 5 Food Industry Research and Development Institute, Hsinchu 300193, Taiwan; 6 Department of Materials Science and Engineering, National Yang Ming Chiao Tung University, Hsinchu City 300093, Taiwan; 7 Graduate Institute of Biomedical Science, China Medical University, Taichung City 406040, Taiwan; 8 School of Dentistry, College of Dental Medicine, Kaohsiung Medical University, Kaohsiung City 813414, Taiwan

**Keywords:** magnetic amphiphilic gelatin nanocarrier, RGD polypeptide, magnetic guidance, articular cartilage regeneration

## Abstract

Osteoarthritis (OA) is a globally occurring articular cartilage degeneration disease that adversely affects both the physical and mental well-being of the patient, including limited mobility. One major pathological characteristic of OA is primarily related to articular cartilage defects resulting from abrasion and catabolic and proinflammatory mediators in OA joints. Although cell therapy has hitherto been regarded as a promising treatment for OA, the therapeutic effects did not meet expectations due to the outflow of implanted cells. Here, we aimed to explore the repair effect of magnetized chondrocytes using magnetic amphiphilic-gelatin nanocarrier (MAGNC) to enhance cellular anchored efficiency and cellular magnetic guidance (MG) toward the superficial zone of damaged cartilage. The results of in vitro experiments showed that magnetized chondrocytes could be rapidly guided along the magnetic force line to form cellular amassment. Furthermore, the Arg-Gly-Asp (RGD) motif of gelatin in MAGNC could integrate the interaction among cells to form cellular stacking. In addition, MAGNCs upregulated the gene expression of collagen II (Col II), aggrecan, and downregulated that of collagen I (Col I) to reduce cell dedifferentiation. In animal models, the magnetized chondrocytes can be guided into the superficial zone with the interaction between the internal magnetic field and MAGNC to form cellular stacking. In vivo results showed that the intensity of N-sulfated-glycosaminoglycans (sGAG) and Col II in the group of magnetized cells with magnetic guiding was higher than that in the other groups. Furthermore, smooth closure of OA cartilage defects was observed in the superficial zone after 8 weeks of implantation. The study revealed the significant potential of MAGNC in promoting the high-density stacking of chondrocytes into the cartilage surface and retaining the biological functions of implanted chondrocytes for OA cartilage repair.

## 1. Introduction

Osteoarthritis (OA) is a common joint disorder characterized by degenerative changes in articular cartilage [1]. Articular cartilage is a compact connective tissue composed of four zones (superficial, middle, deep, and calcified zones) based on the distinct arrangement and organization of chondrocytes and the extracellular matrix (ECM). The superficial zone plays a considerable role in regulating the diffusion transport of nutrients, oxygen, and biomolecules to the underlying cartilage structures [2,3]. More importantly, the superficial zone provides high mechanical strength to protect deeper layers from shear force owing to the tightly arrayed oblate chondrocytes aligned parallel to the cartilage surface. As the articular cartilage is damaged by long-term exposure to risk factors such as older age, sex, and obesity [4], many chondrocytes exhibit necrosis induced by friction in the sunken and unsmooth cartilage surface, particularly in the superficial zone. The biochemical and biomechanical environment of the osteochondral is altered to accelerate pathological changes. Moreover, the self-healing ability of articular cartilage is inadequate because it lacks neural, vascular, and lymphatic systems; therefore, regeneration and repair of articular cartilage pose a challenge for clinical studies. Preliminary clinical strategies conventionally used for treating damaged cartilage include physical rehabilitation, medications, and lubrication substance injections to achieve symptomatic control [5]. However, none of these treatments were able to achieve disease modification.

In the past several years, cell-based therapy has become one of the main strategies for cartilage regeneration. Chondrocytes seeded on biomimetic scaffolds can facilitate cartilage regeneration at damaged sites [6]; however, the repair results have been not evident due to the difficulty in attaching and proliferating implanted cells at the damaged site in the superficial zone, resulting in the surviving chondrocytes being unable to produce enough cartilage-specific ECM to maintain original biomechanical properties [7]. As in cell-based therapy for cellular migration and adhesion during tissue healing, targeted delivery of chondrocytes in the superficial zone is a prerequisite for cartilage regeneration. To achieve accurate control of the implanted chondrocytes to repair the damaged site and effectively maintain the biofunctionality of implanted cells, functional biomaterials combined with cell-based therapy have been developed to provide favorable mechanical 3D environments and improve the activity of implanted chondrocytes and enhance the efficiency of treating OA [8,9]. Nevertheless, there are still certain restrictions on the restoration of a large defective area of OA cartilage. The chondrocytes in normal cartilage tissue are generally stacked in an orderly arrangement to achieve the function of the articular cartilage tissue; however, as the articular cartilage was subjected to degeneration, the surface on the damaged site in the superficial zone gradually lost its functionality and became rough. It will become an important challenge how to guide chondrocytes toward OA articular cartilage repair [10].

Although several reports have mentioned how to improve cell attachment using hydrogel scaffold, the cells are most randomly attached on the surface; therefore, the retention and arrangement of cells delivered with injectable hydrogel at a damaged cartilage site are often limited because of the unstably dynamic motion. In recent years, the use of magnetic force to guide cells has become an important tool for controlling cellular movement for cell and tissue bioengineering [11]. Superparamagnetic iron oxide nanoparticles (SPIONs) have been widely applied in targeting cellular delivery carriers owing to their excellent super-paramagnetism [12,13]; therefore, they can be used to guide implanted cells into damaged sites via an external magnetic field [14]. In addition to fixing the implanted cells on the cartilage defect, the high-density stacking of the cells in the damaged cartilage tissue was also an important concern.

**Scheme 1:**
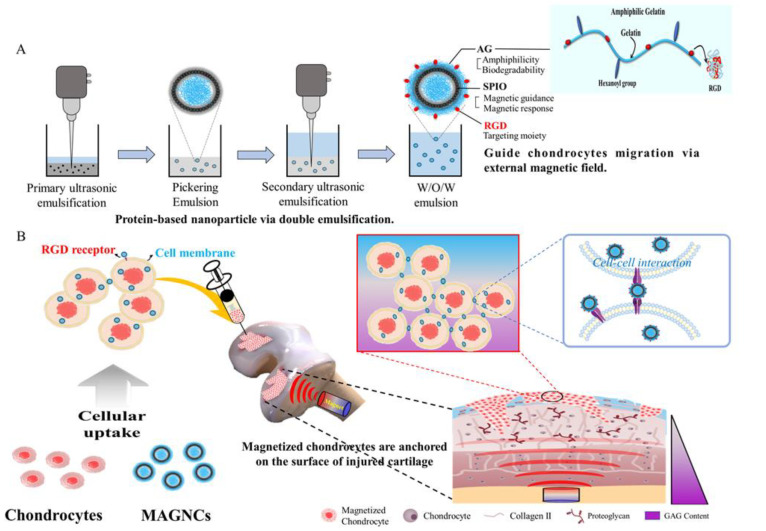
Schematic illustration of (**A**) the synthesis of magnetic amphiphilic-gelatin nanocarriers (MAGNCs) composed of superparamagnetic iron oxide nanoparticles (SPIONs) and amphiphilic gelatin (AG) with RGD to bind and guide chondrocytes; (**B**) magnetized chondrocytes were guided and formed high-density horizontal stacking on the superficial zone of damaged cartilage via internal magnetic force for promoting cartilage repair.

Gelatin is a polypeptide derived from the hydrolysis of collagen; it contains a large number of Arg-Gly-Asp peptides (RGD) that can be used as cellular binding sites for cell adhesion [15]. Gelatin is also an FDA-approved material owing to its biodegradability and biocompatibility. In this study, to investigate whether the directionally high cellular stacking of implanted chondrocytes on the superficial zone can enhance the OA cartilage repair, magnetic amphiphilic-gelatin nanocarrier (MAGNC) was synthesized via double emulsification in the presence of hydrophobic iron oxide nanoparticles. Here, the RGD motif exposed on the MAGNCs acted as a ligand on the implanted chondrocytes toward the damaged site via an internal magnetic field, as shown in Scheme 1A. After co-culture with MAGNCs, the chondrocytes were magnetized via the affinity between MAGNC and cell membrane, and then injected into the arthritis capsule of the OA animal model, as illustrated in Scheme 1B. The chondrocytes were magnetically guided into the damaged cartilage site, where they horizontally stack on the superficial zone of the articular cartilage via an internal magnetic field for OA cartilage repair. The functionality of implanted chondrocytes, including tissue formation and the secretion of cartilage-specific proteins, was also investigated to demonstrate the potential of MAGNCs in cell-based therapy to repair cartilage defects.

## 2. Materials and Methods

### 2.1. Materials

Gelatin from porcine skin, type A (300 bloom, MW: 50k~100k), bovine serum albumin (BSA), hexanoic anhydride, Iron (III) acetylacetonate, 1,2-Hexadecanediod, Oleic acid, Oleylamino, benzyl ether, chloroform, sodium hydroxide, 3-(4,5-Dimethylthiazol-2-yl)-2,5-diphenyltetrazolium bromide (MTT), collagenase II and Alcian Blue 8GX powder were purchased from Sigma-Aldrich (St. Louis, MO, USA). Dimethyl sulfoxide (DMSO) was obtained from Merck (Darmstadt, Germany). F12 medium, fetal bovine serum, trypsin-EDTA, and antibiotics (penicillin/streptomycin, 200 U/mL) were purchased from GIBCO (Grand Island, NY, USA). Total RNA purification mini kit was from FavorPrep, and iScript^TM^ cDNA Synthesis Kit was from Bio-Rad (Hercules, CA, USA). FastStart Universal SYBR Green Master (ROX) was purchased Roche (Mannheim, Germany).

### 2.2. Synthesis of Amphiphilic Gelatin (AG)

Type A 300 bloom Gelatin powder (from porcine skin, 3 g) was dissolved in 40 mL deionized water, and 1 mL of 1N NaOH was added into the gelatin solution with stirring at 70 °C overnight. Ethanol (95%, 30 mL) and hexanoic anhydride (4 mL) were sequentially added to the gelatin solution and stirred for 4 h. The mixed samples were cooled down to room temperature and slowly adjusted to pH = 7.4 with dilute sodium hydroxide. The final solutions were collected in dialysis tubing cellulose membrane with molecular weight cut-off of 14 kDa in ethanol for two days. AG was dried at 60 °C and ground into a powder with a grinding machine and stored in a drying oven at room temperature.

### 2.3. Synthesis of Superparamagnetic Iron Oxide Nanoparticles (SPIONs)

SPIONs were synthesized according to the protocol suggested by Sun et al. [16]. Fe(acac)_3_ (1.059 mg), 1-2-hexadecanediod (3.87 mg), benzyl ether (30 mL), oleic acid (0.9525 mL), and oleylamine (0.9675 mL) were mixed in a 3-neck bottle. Under a nitrogen atmosphere, the mixture was refluxed at 100 °C for 30 min, before being sequentially heated to 200 °C for 1 h and to 285 °C for another 30 min. After being cooled to room temperature, the product was collected via centrifugation at 9000× *g* for 10 min and washed with ethanol 3 times. Subsequently, the SPIONs were redispersed in alcohol under ultrasonic stimulation and stored in ethanol at 4 °C as 10 mg/mL.

### 2.4. Preparation of MAGNCs

MAGNCs were prepared using a double emulsion (W/O/W). AG (0.1 g) was dissolved in 4 mL 0.05 N NaOH at 70 °C and SPIONs (10 mg) were dissolved in 1 mL chloroform. A mixture of 0.6 mL AG solution and 1 mL SPION solution was emulsified for 1 min to obtain a water-in-oil suspension for the first emulsion; subsequently, 2.4 mL of AG solution was added to the mixture and the secondary emulsion was processed for 1.5 min. The mixture was stirred and heated to 60 °C for 40 min to completely remove the chloroform. Finally, the MAGNCs were washed thrice with deionized water and stored in deionized water at 4 °C as 10 mg/mL.

### 2.5. Analysis of MAGNC Characteristics

The morphologies of MAGNCs were analyzed by scanning electron microscope (SEM, SU8000, HITACHI, Tokyo, Japan) and transmission electron microscopy (TEM, Philips CM 200, FEI. Hillsboro, OR, USA). For SEM analysis, MAGNCs were dried in a vacuum desiccator overnight, and then coated with ultrathin platinum layer through platinum sputtering. For TEM analysis, the MAGNC solution was deposited on the carbon-coated copper grid and then the grid was placed in a vacuum desiccator for removing the excess liquid. The particle size of MAGNCs was determined using dynamic light scattering (DLS; Beckman Coulter Delsa™ Nano C particle analyzer, Beckman Coulter, Inc., Brea, CA, USA) equipped with Dual 30 mW laser diodes. Data were analyzed using the CONTIN program (DelsaNano UI software version 3.73/2.30, Beckman Coulter, Inc.). 

### 2.6. Chondrocyte Isolation and Culture

Chondrocytes were harvested from 4-week-old New Zealand rabbits based on a previously described method [17]. The animals were sacrificed by cardiac injection of saturated potassium chloride post gaseous anesthetization with isoflurane. The articular cartilage was collected using a surgical blade and was washed thrice with phosphate-buffered saline (PBS). The cartilage fragment was soaked in 2 mg/mL proteinase (serum-free medium) for 2 h and dissociated in serum-free F12 medium containing collagenase II (2 mg/mL) for 3 h. The chondrocytes were collected at 1500 rpm for 10 min to remove the upper layer of the supernatant, and subsequently mixed with F12 culture medium (2 mg/mL) prior to transferring into 75T cell culture flasks. The isolated chondrocytes were cultured in F12 medium containing 10% bovine serum and antibiotics and incubated at 37 °C in 5% CO_2_. The chondrocytes were used for in vitro and in vivo studies after the second passage.

### 2.7. Cell Viability Test

Chondrocytes were harvested from the 75T culture flask and seeded in 6-well plate at a density of 3 × 10^4^ cells/well. Various concentrations of MAGNCs (0.1, 1, 5, 10, 20, and 50 μg/mL) were incubated with chondrocytes. After 1, 3, and 7-day of incubation, chondrocytes were incubated with a serum-free medium containing MTT (0.5 mg/mL) for 3 h prior to centrifugation at 12,000 rpm for 5 min. After discarding the supernatants, the formazan products were dissolved in 250 μL DMSO by vortexing. The samples were measured at 570 nm using a microplate reader (Spectrostar Nano, BMG Labtech, Offenburg, Germany).

### 2.8. Evaluation of Magnetic-Guiding Chondrocytes

The chondrocytes were cultured with MAGNCs (20 μg/mL) for various time periods. The excess MAGNCs that were not linked to chondrocytes were washed with PBS. The magnetized chondrocytes were stained with calcein-AM and observed under a fluorescence microscope (Oxion Inverso, Euromex, Arnhem, The Netherlands).

### 2.9. N-Sulfated-Glycosaminoglycans (sGAG) Quantification and Alcian Blue Staining

sGAG content was quantified using a Blyscan assay kit (Biocolor Ltd., County Antrim, UK) according to the manufacturer’s instructions. Briefly, the culture medium was collected and mixed with 1 mL dye reagent for 30 min. After the supernatants were removed by centrifuging at 12,000 rpm for 10 min, the samples were mixed with 1 mL dissociation reagent for 20 min and the absorbance was measured at 650 nm using a microplate reader. The sGAG content was calculated from the standard curve. Chondrocytes were incubated overnight in a 1%-Alcian blue solution. After three washes with PBS, the samples were imaged using an optical microscope.

### 2.10. Real-Time Quantitative Polymerase Chain Reaction (RT-qPCR)

The PCR procedure has been described in our previous study [18]. The cell pellets were collected after centrifugation to extract total RNA using a FavorPrep Tissue Total RNA Mini Kit. Using the iScript^TM^ cDNA Synthesis Kit, RNA was reverse-transcribed to cDNA. Finally, cDNA was amplified using SYBR green PCR reagents and the StepOne^TM^ real-time PCR system (Applied Biosystems, Massachusetts, USA). The primer sequences of the target genes are shown in Table S1 (Supplementary Material). *Gapdh* was used as a housekeeping gene. An initial activation step was performed at 95 °C for 20 s followed by 40 cycles at 95 °C for 1 s and 60 °C for 20 s. All reactions were performed in duplicate, followed by a melting-curve analysis for each qRT-PCR run. The relative expression levels were calculated using the 2^−ΔΔ^^*C*_T_^ method.

### 2.11. Animal Study

OA animal models were established according to our previous protocol [17]. All procedures conformed to the guidelines of the Institute of Animal Care and Use Committee of I-Shou University (IACUISU103032). The OA symptoms were induced surgically using anterior cruciate ligament transection (ACLT) after gaseous anesthetization with isoflurane. A sterile magnet (1 mm diameter × 3 mm height) was implanted into the medial femoral condyle. After 6 weeks of surgery, chondrocytes with MAGNCs were injected into the joint capsule to treat OA. The animals were sacrificed after 4 and 8 weeks of injection. The distal femur of the rabbits was extracted after being sacrificed with cardiac injections of saturated KCl solution. The articular tissue was immersed in 4% formaldehyde, then washed three times with PBS, and immersed in decalcifying solution (Decalcifier I, Leica Biosystems, Wetzlar, Germany) until the femur was soft enough to be sectioned. The decalcified tissue was washed with PBS thrice and dehydrated with an ethanol gradient for 20 min at each step and subsequently embedded in paraffin. Sections (7-μm thick) were cut and stained with hematoxylin and eosin (H&E) and Alcian blue.

### 2.12. Immunohistochemical Staining

Double staining was performed with the specific antibodies against Col II (brown) and Col I (green) to investigate their expression and assess their distribution in cartilage tissues. The tissue samples were fixed in 10% paraformaldehyde and decalcified in Decalcifier I prior to embedding in paraffin. The samples were cut into sections of 4 μm thickness. After deparaffinization and rehydration, the tissue slides were blocked in 1% BSA and stained with primary antibodies (collagen II antibody (ARG20787) and collagen I antibody (ARG21965)), followed by incubation with secondary antibodies (goat anti-rabbit IgG-HRP and goat anti-mouse IgG-HRP, respectively). Immunoreactivity was visualized by incubating the sections in DAB and green chromogen buffer.

### 2.13. Simulation of the Magnetic Field

The in vivo three-dimensional magnetic force line produced by the magnet direction in the paramagnetic solution was simulated using COMSOL Multiphysics 5.4 The relative permeability of the paramagnetic solution was taken to be 1.00, corresponding to grade N52-grade neodymium (NdFeB) magnets that were modeled using the remanent flux density of 0.4 tesla, as provided by the manufacturer.

### 2.14. Statistical Analysis

All the data were expressed as mean ± standard deviation (SD). The data were compared by one-way analysis of variance (ANOVA) to evaluate differences among the groups. A difference with *p* < 0.05 was considered statistically significant. *p* < 0.05 (*); *p* < 0.01 (**) and *p* < 0.001 (***). Statistical analyses were performed with GraphPad Prism 8.0 (GraphPad Software, Inc., San Diego, CA, USA).

## 3. Results

### 3.1. Synthesis and Characterization of MAGNCs

**Figure 1:**
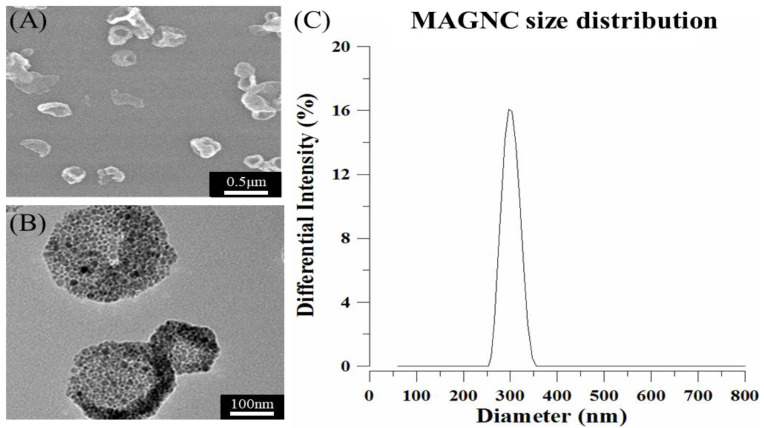
(**A**) SEM image of MAGNC; (**B**) TEM image of MAGNCs; (**C**) particle size of the MAGNCs was ~300 nm, as determined using a DLS instrument.

To anchor the injected chondrocytes on the articular cartilage damaged site via magnetic guidance for cartilage repair, highly uniform SPIONs with an average particle size of 5–7 nm were synthesized, as shown in Figure S1 (Supplementary Data). The SPIONs were hydrophobic owing to the oil phase surfactants used in the manufacturing procedure. To synthesize MAGNCs for providing magnetic cell affinity and guidance, hydrophobic SPIONs were added to the amphiphilic-gelatin (AG) where hexanoic anhydride (HA) molecules were used to substitute the Arg molecules of gelatin. The proton nuclear magnetic resonance spectra in Figure S2 illustrate the primitive and HA-grafted gelatin. There were different chemical shift signals between cardinal and modified gelatin labeled A (2–2.3 ppm) and B (0.9 ppm), which were ascribed to the hexanoyl and methyl group protons from HA, respectively. Moreover, after the reaction of gelatin and HA, the amino groups on the gelatin molecules were substituted by the hexanoyl group on HA. The signal intensity at 2.8 ppm assigned to the primary amino group was significantly reduced from the original site, indicating that the amino group on the original site of gelatin molecules was substituted by the hexanoyl group. MAGNCs were synthesized through a simple double emulsion process in which AG acted as the water phase, and SPIONs as the oil phase to compose the W/O/W (the prepared ratio equaled 0.6:1:2.4) structure. The morphology and particle size of MAGNCs were investigated using microscopy (SEM and TEM) and dynamic light scattering (DLS), respectively (Figure 1). The morphologies of the nanoparticles displayed a collapsed structure with an average particle size of 100~200 nm as shown in SEM and TEM images of Figure 1A,B, respectively. The particle size and polydispersity index of MAGNCs measured by dynamic light scattering (DLS) were estimated around 300 nm and 0.25, respectively, as shown in Figure 1C. The particle size by DLS was much larger than that observed by SEM. Higher hydrodynamic size than primary particle size was also reported in other studies [19,20]. For example, Ahamed et al. reported that hydrodynamic size of pure and Ag-doped (0.5–5%) TiO_2_ NPs measured by DLS was 10–15 times higher than those of sizes calculated from TEM and XRD (primary particle size). Furthermore, MAGNC maintained excellent magnetic response ability as SPIONs, as shown in Figure S3. MAGNCs were well-dispersed in aqueous solution, and no precipitates were observed, suggesting that hydrophobic SPIONs were cladded with hydrophilic AG. In comparison to SPIONs, MAGNCs were relatively stable in the water phase due to gelatin with a large portion of hydrophilic amino acids (lysine, serine, arginine, aspartic acid, and glutamic acid) [21]. According to several reports, the colloidal particle from the green synthesis using nature biomolecules such as fruits, vegetables, or plants provided the various nanoparticles with highly effective biofunctional performance and better cytocompatibility [22,23,24,25]; therefore, MAGNC synthesized from the AG would not affect the native morphology of chondrocytes. In addition, the viability of MAGNC-treated chondrocytes is an essential factor for cell therapy in cartilage repair; therefore, various concentrations of MAGNCs were incubated with chondrocytes for 1, 3, and 7 days to evaluate cytotoxicity. As shown in Figure S4, the chondrocytes incubated with the MAGNCs at 20 μg/mL still exhibited good cell viability after 7 days of incubation, indicating that MAGNCs have negligible cytotoxicity and are compatible with chondrocytes for long-term incubation.

### 3.2. Evaluation of Magnetized Chondrocytes Efficiency

**Figure 2:**
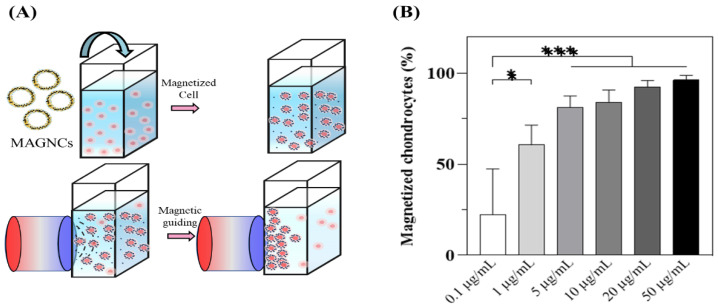
(**A**) Scheme of cell guiding efficiency experiment. (**B**) Evaluation of magnetized chondrocytes at different concentrations of MAGNCs cultured with chondrocytes (*n* = 3). Data represent mean ± SD, * *p* < 0.05, *** *p* < 0.001.

**Figure 3:**
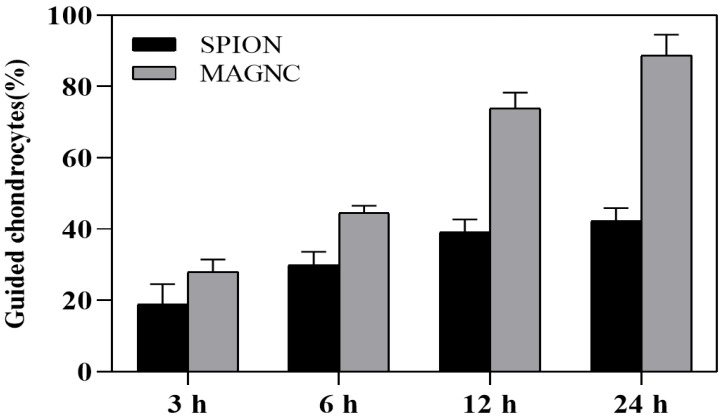
Evaluation of the magnetic-guiding ability of chondrocytes co-cultured with SPIONs and MAGNCs at 20 μg/mL at different time points (*n* = 3).

In this study, magnetized chondrocytes efficiency is a necessary assay to evaluate the cell-guided ability using MAGNC. As shown in Figure 2, different concentrations (0.1, 1, 5, 10, 20, and 50 μg/mL) of MAGNCs were co-cultured with chondrocytes. When the concentration of MAGNC was less than 10 μg/mL, the magnetized chondrocytes were approximately 82%; however, upon increasing the concentration of MAGNC to 20 μg/mL or more, the magnetized cells approached 100% in the range of 20–50 μg/mL; therefore, we used 20 μg/mL of MAGNC as the experimental concentration for the minimum dosage with the maximum guiding ability (95.6% of guided cells). In addition to the concentration of MAGNC, a time-dependent manner of intracellular MAGNC also played an important role in this study. As shown in Figure 3, the magnetized chondrocytes through SPIONs uptake of chondrocytes appeared at 3 h and increased to 12 h, but no further increase over 12 h. In contrast, using MAGNCs, the chondrocytes were not only magnetized by the cell uptake of the MAGNCs but also the RGD ligands on the MAGNCs can increase the interaction between MAGNCs and chondrocytes; therefore, the number of magnetized cells using MAGNCs presented a continuous increase over time, even after 12 h of incubation, which is mainly sourced from the interaction between RGD and cell membrane.

### 3.3. Magnetic Guidance of Cells

**Figure 4:**
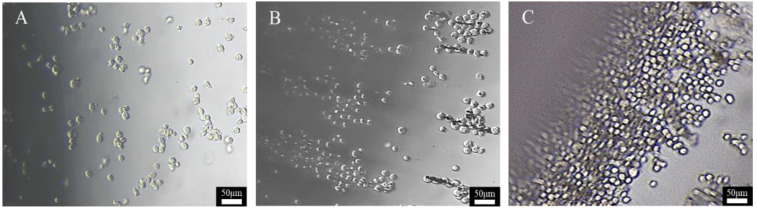
Distribution and movement of chondrocytes under applied magnetic field. Chondrocytes were incubated with different magnetic nanoparticles at 20 µg/mL and analyzed after 12 h of incubation on an inverted microscope for (**A**) chondrocytes alone, (**B**) chondrocytes treated with SPIONs, and (**C**) magnetized chondrocytes using MAGNCs.

The guiding efficiency of intra-articularly injected implanted chondrocytes is important for the cell therapy of OA. To evaluate the magnetic field distribution in in vitro cell culture, we used COMSOL Multiphysics 5.4 to simulate the magnetic field line of the external magnet. As shown in Figure S5, the magnetic field lines in all directions point toward the magnet that was placed under the culture dish, implying that the cells with SPION or MAGNC would be affected by the external magnetic field along the magnetic field lines to concentrate on the magnet position. The migration behavior of chondrocytes with SPION treated toward the magnet was demonstrated in Movie S1. Furthermore, the magnetized chondrocytes showed rapid movement toward the magnet as applied with a magnetic field, as shown in Movie S2. The chondrocytes alone groups without MAGNCs and external magnet could not form the cell stacking depicted in Figure 4A, whereas SPION treated chondrocytes could be guided into the specific area under an external magnetic field (Figure 4B). Although the results demonstrated that both MAGNC- and SPION treated cells could be magnetically guided to form cellular migration along with the magnetic field distribution, high-density cell stacking was observed in MAGNC compared to the SPION group, as shown in Figure 4C, indicating that MAGNCs with SPIONs inside the AG shell and RGD exposure could significantly enhance the rapid motion and aggregation of chondrocytes compared to SPION alone. More importantly, magnetized chondrocytes displayed layer-like horizontal stacking via the synergy of RGD binding and magnetic navigation, while SPION treated cells were sparsely dispersed without clear horizontal stacking, indicating that gelatin of MAGNCs not only increased the internalization of magnetic nanoparticles, but also enhanced the cell association via RGD-cell interaction. In addition to the magnetic guidance evaluation, post-incubation magnetized chondrocytes were also observed with an external magnet. As shown in Figure S6, all groups showed good cell viability. In order to further observe the dynamic cell movement under the interaction between magnetized chondrocyte and magnetic field, we constructed a structure with a tapering gap using two pieces of silicon-rubber hydrogel and placed a magnet near the tip of the gap to form a magnetic field. Subsequently, magnetized chondrocytes were placed at the other side for simulating the migration and stacking of magnetized chondrocytes toward the damaged site under a magnetic field. As shown in Figure S7, without a magnetic field, only a few magnetized chondrocytes could arrive at the gap tip. In contrast, the chondrocytes could be effectively and rapidly migrated towards the damaged area under the influence of an external magnetic field. More importantly, the magnetized chondrocyte would further move into the crevices at the damaged region. After 30 min, it was found that the cells were not only still maintained at the crevices but also formed high-density lateral stacks on the surface of the hydrogel. It is worth mentioning that some chondrocytes still retained in the gap when the magnet was removed after the magnetized chondrocytes were magnetically moved into the gap; however, the chondrocytes on the surface of the damaged region would be gradually scattered, resulting in a very sparse distribution of cells in 30 min. 

### 3.4. sGAG Content Assay

**Figure 5:**
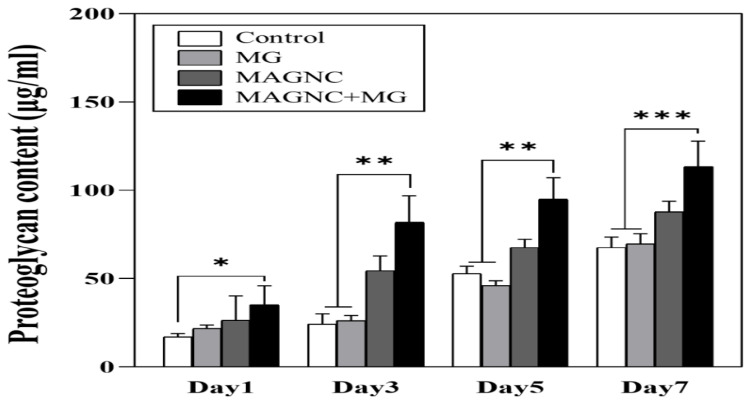
sGAG secretion in cultures with different stimulations (MG, MAGNCs, and MAGNCs+MG) at 1, 3, 5, and 7 days (*n* = 3). Data represent mean ± SD, * *p* < 0.05, ** *p* < 0.01, *** *p* < 0.001.

sGAG is one of the major components of the ECM in hyaline cartilage and has been considered a marker of chondrogenic differentiation. Without any treatment, the lowest sGAG content secreted from chondrocytes was measured, as shown in Figure 5. In contrast, as the chondrocytes were magnetized by MAGNC and treated under magnetic guidance (MG), the intensity of sGAG content in the MAGNC+MG group was much higher than that in other groups. The increased sGAG may be attributed to the aggregation of the magnetized chondrocytes, according to the reports that cell culture in aggregation form would favor the maintenance of the chondrocyte phenotype and exhibit positive effects on the chondrogenic differentiation [26,27]. Moreover, the sGAG stain in the MAGNC+MG group increased with time, with the greatest intensity appearing at day 7 (Figure S8), suggesting that the magnetized chondrocytes under magnetic field exhibited remarkable chondrogenic differentiation.

### 3.5. RT-qPCR

**Figure 6:**
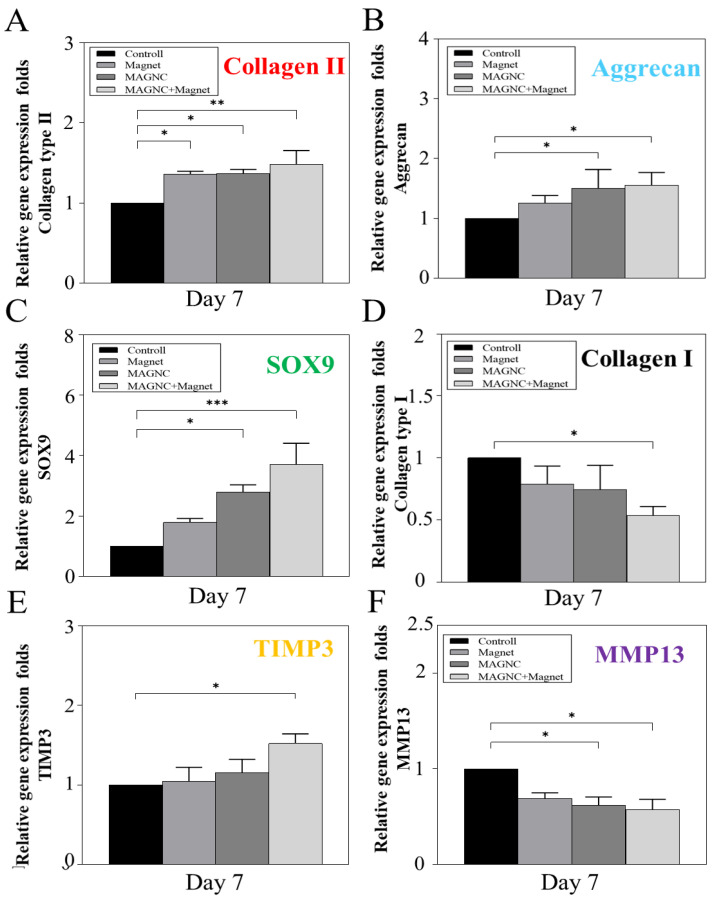
Relative expression levels of cartilage-specific and matrix degradation genes, including (**A**) Col II, (**B**) aggrecan, (**C**) SOX9, (**D**) Col I, (**E**) TIMP3, and (**F**) MMP13. Data represent mean ± SD (*n* = 3), * *p* < 0.05, ** *p* < 0.01, *** *p* < 0.001.

To evaluate the chondrogenic biofunctionality under the magnetic-guiding effect, the expression levels of six types of cartilage-related genes, including collagen type II (Col II), aggrecan (Agg), SOX9, TIMP3, collagen type I (Col I), and MMP13, were quantified using RT-qPCR for the chondrocytes treated with MAGNC, MG, or MAGNC+MG for 7 days. Col II and Agg are the predominant ECM components of articular cartilage and chondrogenic-related markers [28,29]. As shown in Figure 6A,B, the expression of the chondrogenic-related marker genes Col II and Agg was higher in the MAGNC+MG group than in the other groups. SOX9 is a key transcription factor involved in chondrogenic differentiation [30,31]. The gene expression of SOX9 (Figure 6C) was also upregulated by 3.7-fold in the MAGNC+MG group than in the control group, thus indicating a significant enhancement in the expression of chondrogenic-specific genes in the MAGNC+MG group. The phenomenon of chondrocyte dedifferentiation and biofunctionality deactivation has been a major hurdle in the development of cell therapies. It is worth mentioning that in Figure 6D, chondrocytes treated with MAGNC+MG exhibit significantly decreased Col I gene expression, which has been regarded as a dedifferentiation marker [32]. Moreover, MMP13 digests ECM molecules, including cartilage Col II and proteoglycan, whereas TIMP3 inhibits MMP13 by forming high-affinity complexes [33,34]. As shown in Figure 6E,F, chondrocytes treated with MAGNC+MG showed increased expression of TIMP3 and decreased expression of MMP13, indicating that treatment with MAGNC+MG can downregulate the gene expression of MMP13 and further prevent the degradation of ECM components. These results likely reflect that GAG synthesis was the highest in the MAGNC+MG group (Figure 5). The above results revealed that MAGNCs served as magnetic navigation while enhancing the differentiated phenotype and biofunctionality of magnetized chondrocytes.

### 3.6. Animal Study

**Figure 7:**
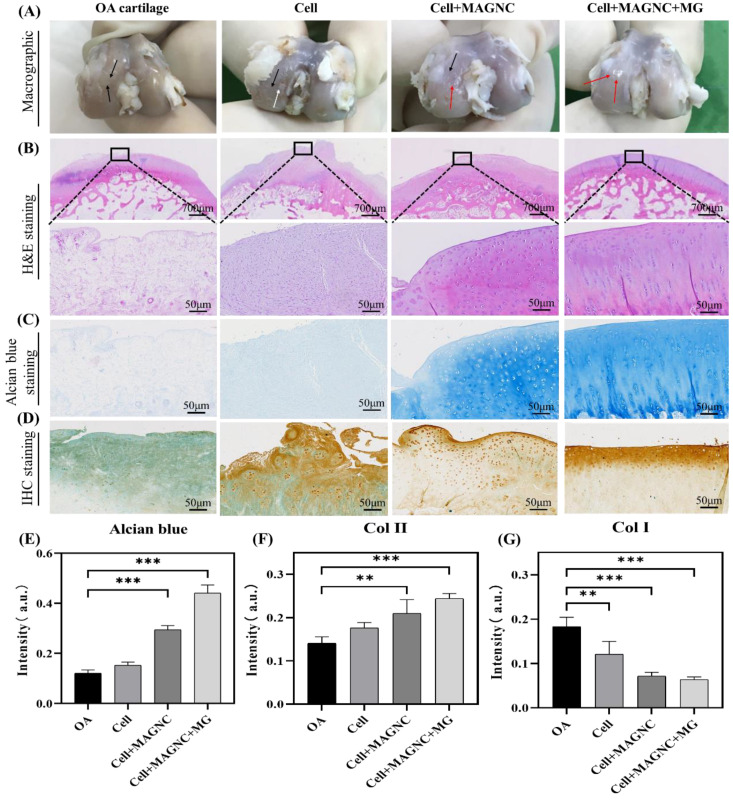
Macrographic observation and histological evaluation of magnetic-guiding outcome in the articular cartilage of OA animal model at 8 weeks post intra-articular injection. (**A**) Macrographic observation following treatment with Cell, Cell+MAGNC, or Cell+MAGNC+MG; (**B**) H&E staining of articular cartilage repairing sections following treatment with Cell, Cell+MAGNC, or Cell+MAGNC+MG with magnetic guidance 8 weeks post- osteoarthritis; (**C**) Evaluation of sGAG staining cartilages with Alcian blue following treatment with Cell, Cell+MAGNC, or Cell+MAGNC+MG with magnetic guidance 8 weeks post-osteoarthritis; (**D**) immunohistochemistry for Col I and II to observe the pathological and the repaired behavior via MAGNC, MG, and MAGNCs/MG in tandem with cell therapy at week 8; (**E**) optical density analysis of Alcian blue staining, immunohistochemistry for (**F**) Col II and (**G**) Col I. Data represent mean ± SD, ** *p* < 0.01, *** *p* < 0.001.

To characterize the in vivo magnetic field distribution, COMSOL Multiphysics 5.4 was used to simulate the different directions of the implanted magnetic force. As shown in Figure S9, the magnet was implanted into the medial femoral joint, where the magnetic field could penetrate the cartilage surface to focus the magnetic force on the damaged site; therefore, we could use the simulation model to explore the cell distribution under an applied magnetic field. To evaluate the combined effect of MAGNCs with MG to assist cell therapy for in vivo cartilage repair, the osteoarthritis in an animal model was established through ACLT surgery. The magnet was implanted into the medial femoral joint to guide the vertical magnetic field through the cartilage surface, as shown in Figure S10. Magnetized chondrocytes were injected intra-articularly 6 weeks after ACLT surgery. Histological staining assays were performed to evaluate the therapeutic effects of MAGNCs on OA. As shown in Figure 7A, in the cell group, the cartilage surface in the OA group was the roughest at 8 weeks (black arrows), and the new white granulation tissue was observed (white arrows). There are still certain limitations on the efficacy of degenerative arthritis, as cells in the damaged site cannot be fixed. Although the Cell+MAGNC group also exhibited a discontinuity boundary, the degree of cartilage repair was significantly better than that of the OA group. Compared with other groups, the cartilage surface in the Cell+MAGNCs+MG group appeared smooth and flat (red arrows) similar to native tissue, indicating that MAGNCs provided a magnetic response ability for magnetized chondrocytes to securely fix the injected chondrocytes on the cartilage surface to form orderly stacking, which promoted the secretion of ECM components for implanted chondrocytes to increase the repair efficacy.

To further investigate the recovery of cartilage defects, cartilage tissues were stained with H&E and Alcian Blue. As shown in Figure 7B, the tissue surface of the OA group was unsmooth and defective after 8 weeks. There was a large amount of irregular newborn tissue on the surface in the cell group; however, without the application of a magnetic field, the chondrocytes in the Cell+MAGNC group were not easily and stably anchored on the rough surface of the damaged cartilage, leading to the formation of a slightly proliferative layer on the cartilage surface. Conversely, in the Cell+MAGNC+MG group, the implanted cells were magnetically guided and well-stacked, leading to an orderly arrangement of chondrocytes in the repaired cartilage region, similar to native tissue, as shown in Figure 7B. Furthermore, as illustrated in Figure 7C, sGAG was secreted and precipitated in the depth layer in the Cell+MAGNC+MG group, which corresponded to the results of H&E staining, indicating that the repair effect is closely related to the orderly cell stacking derived from the interaction of nanocarriers and cells via RGD under magnetic guidance.

Col II is one of the major components of the hyaline cartilage matrix, used as a cartilage repair marker to identify the regeneration effectiveness [28], whereas Col I is used as a fibrocartilage marker to confirm the cartilage degeneration degree [32]. Both were verified by immunohistochemical analysis (Figure 7D). At 8 weeks, in the OA group, Col I was clearly observed in cartilage tissue and Col II was the lowest, indicating that the degenerated cartilage model was successfully induced. At 8 weeks after implantation in the cell group, fibrous tissue on the newborn cartilage was observed and displayed less Col II staining; however, Col II was locally secreted on the cartilage surface in the MAGNC+Cell group, indicating that MAGNC could significantly promote the proliferation and differentiation of implanted cells on the damaged surface. Moreover, in the Cell+MAGNC+MG group, Col II level was the highest on the cartilage surface, indicating that high-density cells were stacked via cell/RGD-MAGNC/cell interaction by magnetic guidance. Consequently, the production of Col II was enhanced to develop biofunctional hyaline cartilage for OA cartilage repair. The intensities of sGAG and Col II in the Cell+MAGNC+MG group were higher than those in the other groups (Figure 7E,F). Col I level in the Cell+MAGNC+MG group was the lowest among all groups (Figure 7G), which was consistent with the results of RT-qPCR (Figure 6D). The in vivo study demonstrated that the synergy of MAGNC carriers and magnetic navigation not only promoted the orderly stacking of the chondrocytes into the cartilage surface, but also retained the biological functions of implanted chondrocytes.

## 4. Discussion

Cell therapy, which uses an intra-articular injection to deliver implanted cells into cartilage defects, has been considered as a potential treatment for cartilage regeneration; however, not all injected cells are stably anchored to the surface of the cartilage, thus reducing the effectiveness of cell therapy for cartilage regeneration [6]. In recent years, several studies have indicated that magnetic targeting delivery technology (MTDT) can be used for cell manipulation using magnetic particles such as SPIONs [35]. Although several surface coating methods have been used to promote the interaction between SPIONs and cells [36,37], there are still shortcomings with the above coating procedure, such as complicated coating and processing control [38]. In contrast, the RGD motif of gelatin could provide a cellular binding site to increase the interaction with cells via RGD binding and cell adhesion. Furthermore, MAGNC nanoparticles with SPION enrichment exist in the shell to increase the magnetic force and provide a larger contact area, benefiting from the increased interaction with the cell membrane [39]; therefore, MAGNC nanoparticles were designed to modulate chondrocyte behavior and increase the interaction between the carriers and cells. As illustrated in Figure 3, compared to the SPION group, the MAGNC group revealed significantly higher magnetized cells, indicating that the RGD motif on MAGNCs can act as a ligand to enhance the cell association with chondrocytes [40,41].

The anchoring ability of injected chondrocytes to the cartilage surface is also a key factor for the regeneration of cartilage and is strongly influenced by magnetic force, magnetic field direction, and adhesive friction [42,43,44,45]; however, some of these details are difficult to be quantified in vivo or are not yet known. Based on the directional arrangement of chondrocytes in articular cartilage tissue, we further designed the MAGNC with magnetic-guiding ability to guide more chondrocytes for overcoming the condition of low cellular retention. The result in Figure S7 demonstrated that a large number of magnetized chondrocytes could be guided into the tip gap with the external magnetic field to simulate in vivo guiding of magnetized chondrocytes in cartilage damaged sites. The high-density magnetized chondrocytes remained in the damaged crevice, and then produced a horizontal stacking of the cells on the surface. The results demonstrated that the RGD motifs on the MAGNC can increase cell binding, and the magnetized cells can simultaneously produce obvious stacking and arrangement in an incubation time less than 12 h under the action of an external magnetic field.

In this in vivo study, osteoarthritis was surgically induced by anterior cruciate ligament transection (ACLT) and partial medial meniscectomy on one knee of male New Zealand rabbits. Subsequently, the magnet was implanted into the medial femoral to simulate the magnetic field perpendicular to the cartilage surface for investigating its cellular guiding ability into the cartilage damaged site. The animal model results further confirmed that implanted chondrocytes alone could not be stably anchored on the damaged site without integrating MAGNCs and MG. In addition, the surviving chondrocytes would irregularly hyperplasia on the cartilage surface, limiting the effectiveness of cartilage repair. In contrast, the implanted chondrocytes engrafted with magnetic MAGNCs could be effectively guided toward the damaged cartilage and formed the high-density stacking on the superficial zone for OA cartilage repair by an internal magnetic field. 

The retention of implanted chondrocytes on the superficial zone of the cartilage could promote the secretion of cartilage-specific ECM molecules such as proteoglycans (Figure S8) and enhance the smoothness of the articular surface, as depicted in Figure 7B. In this study, although the magnet must be implanted via an invasive procedure, requiring a recovery period for the incision stage, the implanted magnet could provide a stable magnetic force surrounding the cartilage surface (as shown in Figure S9) in large region defects of cartilage. In contrast, an external magnetic device not only required an additional fixation device after targeting but also needed a larger and more precise magnetic force to guide the chondrocytes, which is very challenging and difficult to achieve. In summary, the above results have demonstrated that an all-in-one implanted magnetic MAGNC system can perform the targeting and fixation of magnetized chondrocytes for promoting cartilage repair of an OA articular cartilage.

## 5. Conclusions

In summary, we successfully designed a MAGNC synthesized from HA-grafted gelatin and SPION. MAGNC could anchor the interaction of chondrocyte cells via the RGD motif of gelatin to increase the number of magnetized cells. Most importantly, the magnetized chondrocytes exhibited excellent magnetic-guiding characteristics and were magnetically guided to the damaged cartilage site to form a high-density arrangement by magnetic force. High-density cellular stacking could significantly upregulated Col II gene expression to improve cartilage repair. Furthermore, the in vivo results indicated that the implanted chondrocytes with MAGNCs could enhance the repair of degenerated cartilage to achieve full closure of the lesion with a smooth surface under magnetic cell delivery. The development of MAGNCs in magnetized chondrocyte and articular cartilage tissue engineering has great potential for further applications.

## Data Availability

Not applicable.

## Supplementary Materials

The following supporting information can be downloaded at: https://www.mdpi.com/article/10.3390/polym14040809/s1, Figure S1: TEM image of SPIONs; Figure S2: ^1^H-NMR spectrum of gelatin molecules in D_2_O: (A) primitive and (B) modified gelatin molecules; Figure S3: Evaluation of magnetic-guiding ability of the nanoparticles (A) blank, (B) SPIO, (C) MAGNC suspensions (20 μg/mL MAGNCs were homogeneously dispersed in d^2^H_2_O), (D) photo magnetic-guiding test of SPIO nanoparticles, (E) magnetic-guiding test of MAGNCs; Figure S4: Cell proliferation assessment: chondrocytes were co-cultured with MAGNCs containing different concentrations of SPIONs for 1, 3, and 7 days. Data represent mean ± SD, * *p* < 0.05 ** *p* < 0.01, *** *p* < 0.001; Figure S5: Simulation illustration of in vitro magnetic flux density distribution; Figure S6: (A) Illustration of the in vitro magnetic guidance of MAGNC-labeled chondrocytes. (B) Florescence microscopy of the cells around magnetic enrichment with magnitudes of 10× (scale bar = 100 μm); Figure S7: In vitro experiment to simulate cell migration by an external magnetic force to observe the dynamic movement of chondrocytes; Figure S8: Alcian blue staining. GAGs secreted from chondrocytes were stained with Alcian blue to demonstrate the significant differences between GAGs and chondrocytes receiving various stimulations. (Scale bar: 200 μm); Figure S9: Simulation illustration of in vivo magnetic flux density distribution. Magnetic flux density distribution in (A) the x-y, (B) x-z, and (C) y-z planes along the cartilage surface. Magnetic flux density distribution in the (D) x-y (E) x-z and (F) y-z planes along magnet in the femur to simulate external magnetic field through cartilage surface; Figure S10: Location of the magnetic column. (A) X-ray images of implanted magnetic column; top view; (B) side view; Table S1: Primers used in this study; Movie S1: Observation of the guidance of SPIO magnetized chondrocytes via external magnet; Movie S2: Observation of the guidance of MAGNC-magnetized chondrocytes via external magnet.
