# Supplementary material for: High-Density Horizontal Stacking of Chondrocytes via the Synergy of Biocompatible Magnetic Gelatin Nanocarriers and Internal Magnetic Navigation for Enhancing Cartilage Repair
Source: Polymers (Basel). 2022 Feb 19;14(4):809. doi: 10.3390/polym14040809 (PMC8963011; doi:10.3390/polym14040809)
Supplement: Supplementary file 1 [file polymers-14-00809-s001.zip › polymers-1544024- Supplementary file.pdf]

Supplementary Materials

High-density Horizontal Stacking of Chondrocytes via the Synergy of Biocompatible Magnetic Gelatin Nanocarriers and Internal Magnetic Navigation for Enhancing Cartilage Repair

Shan-Wei Yang[a1], Yong-Ji Chen[b1], Huai-En Lu[c], Ching-Jung Chen[d], Chin-Yi Yang[b], Jen-Hao Tsai[b], Jen Tsai Liu[e*], San-Yuan Chen[f,g,h*], Shwu-Jen Chang[b*]

[a] Department of Orthopedics, Kaohsiung Veterans General Hospital, Kaohsiung City, 813414, Taiwan

[b] Department of Biomedical Engineering, I-Shou University, Kaohsiung City, 82445, Taiwan

[c] School of Opto-Electronic Technology, University of Chinese Academy of Sciences, Beijing, 100049, PR China

[d] College of Materials Science and Opto-Electronic Technology, University of Chinese Academy of Sciences, Beijing, 100049, PR China

[e] Food Industry Research and Development Institute, Hsinchu, 300193, Taiwan

[f] Department of Materials Science and Engineering, National Yang-Ming Chiao Tung University, Hsinchu, 30010, Taiwan

[g] Graduate Institute of Biomedical Science, China Medical University, Taichung City, 406040, Taiwan

[h] School of Dentistry, College of Dental Medicine, Kaohsiung Medical University, Kaohsiung, 807, Taiwan

Corresponding author

[*]E-mail addresses: sjchang@isu.edu.tw (S.-J Chang)

[*]E-mail addresses: sanyuanchen@nctu.edu.tw (S.-Y. Chen)

*E-mail addresses: alpharoger@gmail.com (H.-E. Lu)

[1] These authors contribute equally to this work.

Supplementary data

Table S1. Primers used in this study.

Marker gene	Sequences (5'-3')		Accession No.
Gapdh	Forward	AGGTCATCCACGACCACTTC	NM_001082253
	Reverse	GTGAGTTTCCCGTTCAGCTC	
Col II	Forward	CAACAACCAGATCGAGAGCA	NM_001195671
	Reverse	GCTCCACCAGTTCTTCTTGG	
Agg	Forward	GACTCTTTCACATGCTTATGCCTTCC	XM_002723376
	Reverse	GTGACGATGCTGCTCAGGTGTG	
SOX9	Forward	TTCATGAAGATGACCGACGA	AY_598935
	Reverse	GTCCAGTCGTAGCCCTTGAG	
TIMP3	Forward	CACCGTCAAGCAGATGAAGA	NM_001195682
	Reverse	CACAGCCCCGTGTATACCTT	
Col I	Forward	TAAGAGCTCCAAGGCCAAGA	NM_001082253
	Reverse	TGTTCTGAGAGGCGTGATTG	
MMP13	Forward	TCCCTGGGTCTGTTTCACTC	NM_001082037
	Reverse	TCTCCCCTCAGAGTGCTGAT	

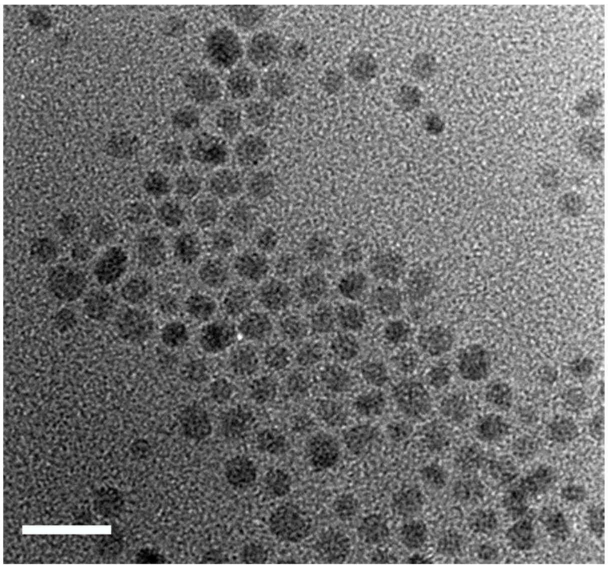

Figure S1. TEM image of SPIONs; scale bar = 20 nm.

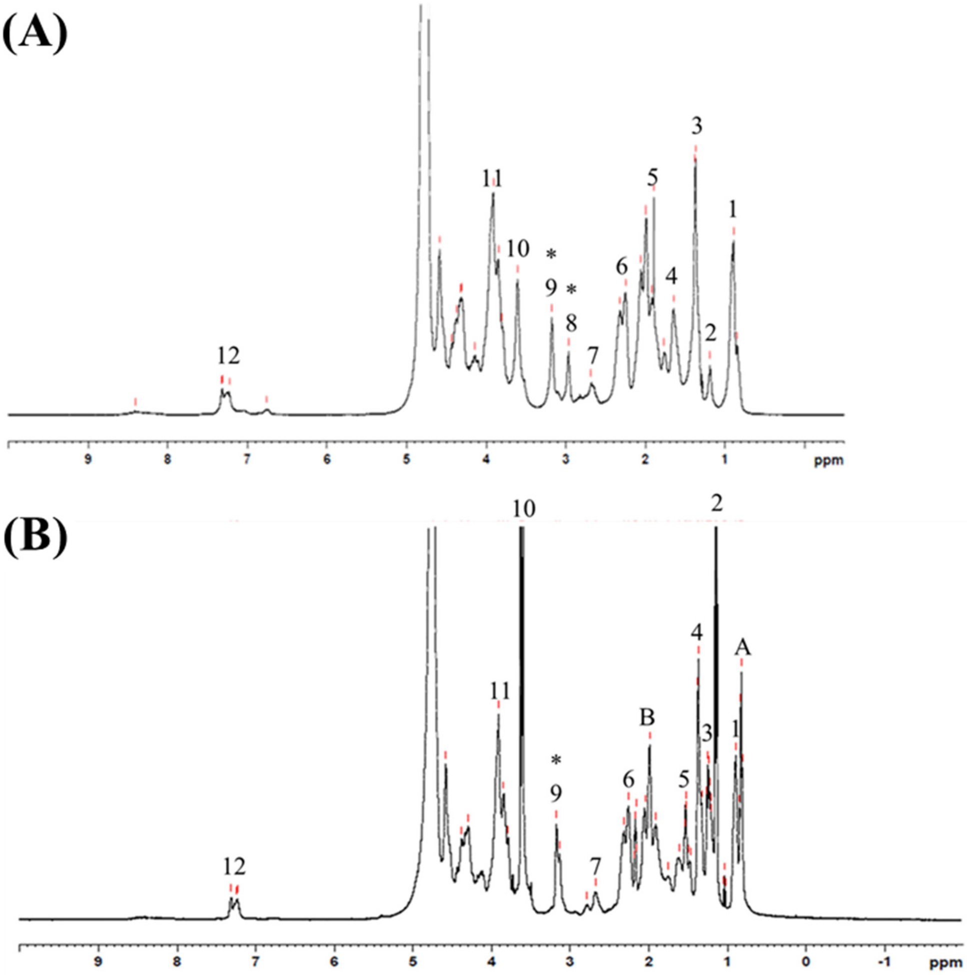

Figure S2. ^{1}H-NMR spectrum of gelatin molecules in D_2O: (A) primitive and (B) modified gelatin molecules.

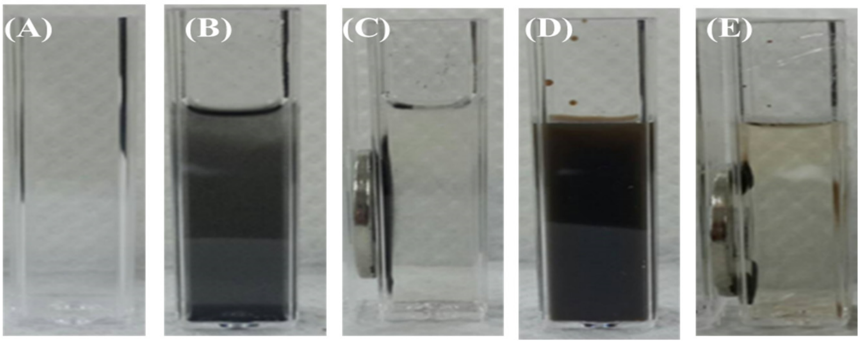

Figure S3. Evaluation of magnetic-guiding ability of the nanoparticles (A) blank, (B) SPIO, (C) MAGNC suspensions (20 µg/ml MAGNCs were homogeneously dispersed in d^2H_2O), (D) photo magnetic-guiding test of SPIO nanoparticles, (E) magnetic-guiding test of MAGNCs.

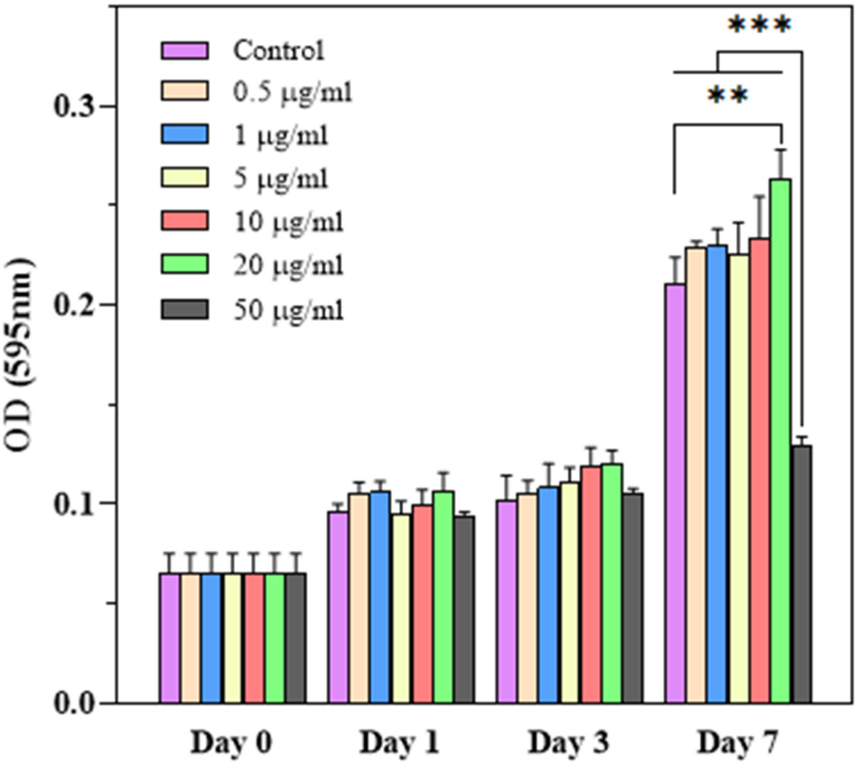

Figure S4. Cell proliferation assessment: chondrocytes were co-cultured with MAGNCs containing different concentrations of SPIONs for 1, 3, and 7 days. Data represent mean±SD, * $p < 0.05$ ** $p < 0.01$, *** $p < 0.001$.

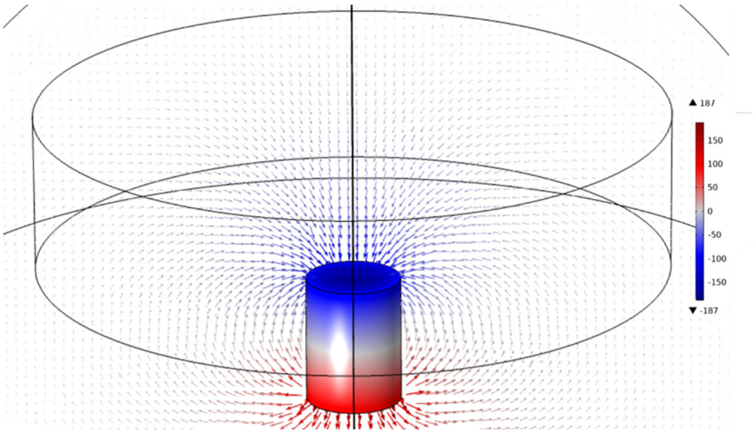

Figure S5. Simulation illustration of in vitro magnetic flux density distribution.

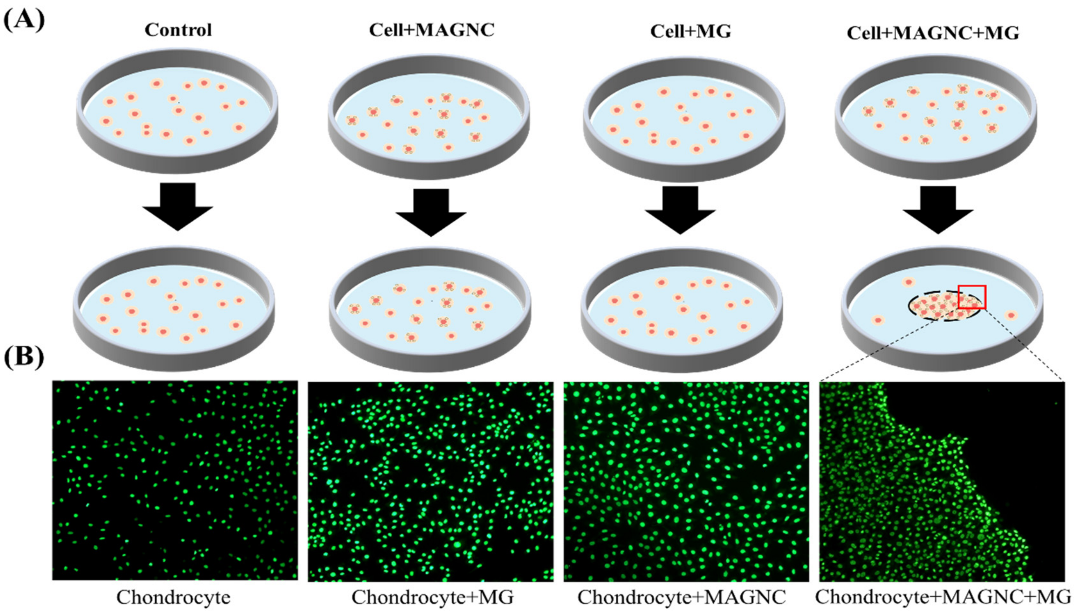

Figure S6. (A) Illustration of the in vitro magnetic guidance of MAGNC-labeled chondrocytes. (B) Florescence microscopy of the cells around magnetic enrichment with magnitudes of 10× (scale bar = 100 μm)

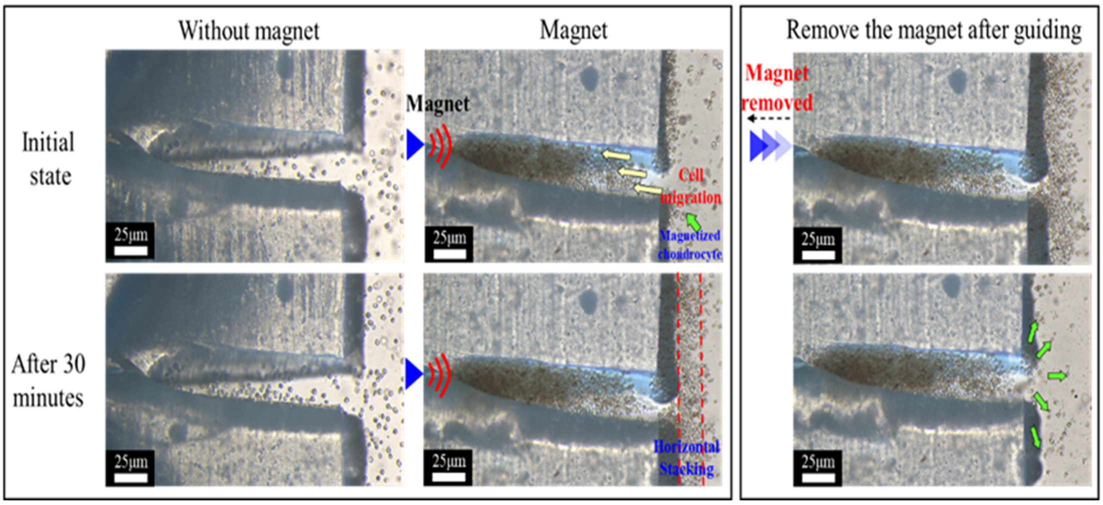

Figure S7. In vitro experiment to simulate cell migration by an external magnetic force to observe the dynamic movement of chondrocytes.

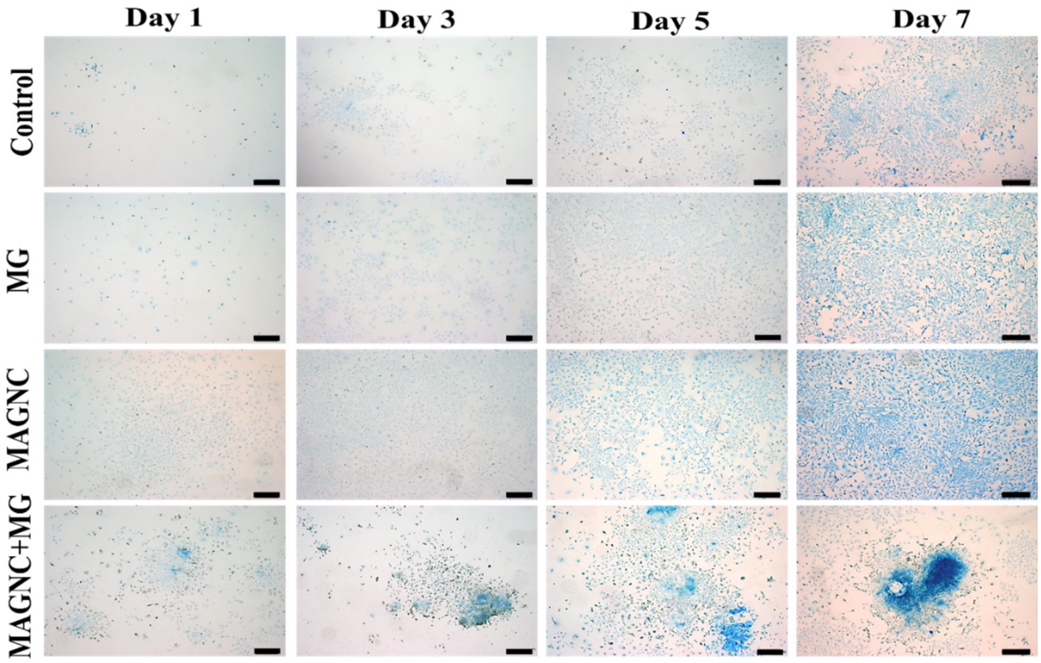

Figure S8. Alcian blue staining. GAGs secreted from chondrocytes were stained with Alcian blue to demonstrate the significant differences between GAGs and chondrocytes receiving various stimulations. (Scale bar: 200 μm)

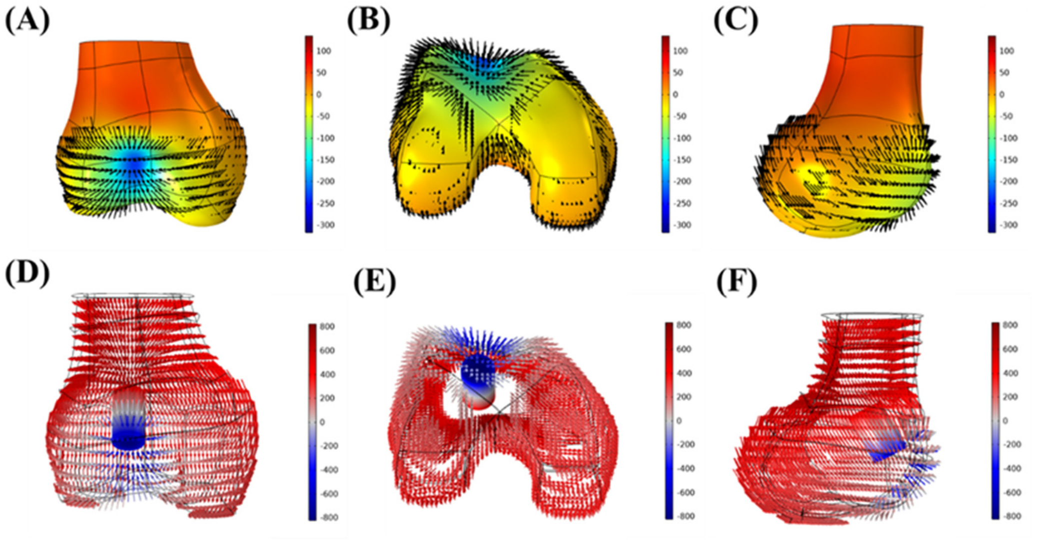

Figure S9. Simulation illustration of in vivo magnetic flux density distribution. Magnetic flux density distribution in (A) the x-y, (B) x-z, and (C) y-z planes along the cartilage surface. Magnetic flux density distribution in the (D) x-y (E) x-z and (F) y-z planes along magnet in the femur to simulate external magnetic field through cartilage surface.

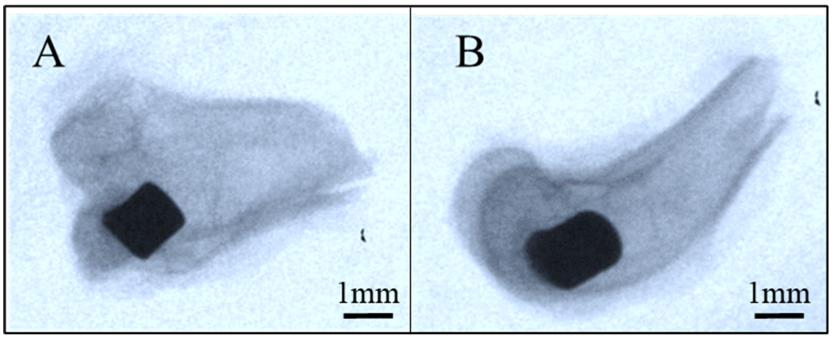

Figure S10. Location of the magnetic column. (A) X-ray images of implanted magnetic column; top view; (B) side view.

Movie S1. Observation of the guidance of SPIO magnetized chondrocytes via external magnet.

Movie S2. Observation of the guidance of MAGNC-magnetized chondrocytes via external magnet.
